# F127. GLOBAL RECOVERY IN A FIRST EPISODE PSYCHOSIS PROGRAM IN SOUTH AMERICA

**DOI:** 10.1093/schbul/sby017.658

**Published:** 2018-04-01

**Authors:** Barbara Iruretagoyena, Nicolas Crossley, Juan Undurraga, Carmen Castañeda, Carlos Gallardo, Felipe Mancilla, Alfonso Gonzalez

**Affiliations:** 1 P. Universidad Catolica de Chile; 2 Early Intervention Program, J. Horwitz Psychiatric Institute

## Abstract

**Background:**

Metanalisis show that global recovery, (a state of clinical and social well functioning) is achieved by 13.5% patients (25%-75% quartiles 8.1–20%) (Jääskeläinen, 2013) diagnosed with schizophrenia. It has also been suggested that recovery is higher in low or lower middle-income countries compared to high and upper middle income countries. However, this is only based in a few studies. We here looked at the number of patients with first episode psychosis that met recovery criteria based on both clinical and social domains in a South American early intervention sample. We also examined whether recovery was associated with factors such as diagnoses, sex, education, substance use and duration of untreated psychosis.

**Methods:**

This is a cross-sectionall study in an outpatient First Episode Psychosis program in Chile. We gathered information on different aspects of the patients, including sociodemographic, clinical, functional and metabolic status. FAST (Functional Assessment Short Test) and SS-DSM5 (Symptom Severity Scale of the DSM5 for Schizophrenia) were applied to patients. Global recovery was defined as the presence for at least 6 months of: 1. Working or studying. 2. SS-DSM5 scale with no dimension with score over two. 3. FAST with score under 21 (which correlates with GAF > 61). The group who met recovery criteria (improvement in both clinical and social domains) was identified, and correlation and regression analysis were performed to explore the association between global recovery and selected variables.

**Results:**

We included 80 patients in this study. Overall, 20% met global recovery criteria. Patients who did not accomplish recovery did so because of being unemployed (80.6%), not studying (79.7%), or scoring above threshold in SS-DSM5 cognitive (54.7%) and negative (54.7%) symptom domains. Univariate correlation analyses showed a significant association of global recovery with recreational drug use, diagnoses, and duration of untreated psychosis (all corrected for multiple comparisons). After multiple regression analysis including these variables, age and gender, the only one associated with recovery was shorter duration of untreated psychosis (p=0.02) OR 0.616 (IC95% 0.409–0.925).

**Discussion:**

The number of patients achieving global recovery is consistent with the one reported for schizophrenia in previous meta-analysis and with studies on recovery after first episode psychosis (16.6% (25%-75% quartiles 9–20.4)) (Jääskeläinen, 2013). Negative and cognitive symptoms frequently impair patient recovery. On the other hand the duration of untreated psychosis shows itself as one of the most important characteristics related with functional prognoses.

